# Oligomerization as a strategy for cold adaptation: Structure and dynamics of the GH1 β-glucosidase from *Exiguobacterium antarcticum* B7

**DOI:** 10.1038/srep23776

**Published:** 2016-03-31

**Authors:** Leticia Maria Zanphorlin, Priscila Oliveira de Giuseppe, Rodrigo Vargas Honorato, Celisa Caldana Costa Tonoli, Juliana Fattori, Elaine Crespim, Paulo Sergio Lopes de Oliveira, Roberto Ruller, Mario Tyago Murakami

**Affiliations:** 1 Brazilian Bioethanol Science and Technology Laboratory, Campinas, São Paulo, Brazil; 2 Brazilian Biosciences National Laboratory from the National Center for Research in Energy and Materials, Campinas, São Paulo, Brazil

## Abstract

Psychrophilic enzymes evolved from a plethora of structural scaffolds via multiple molecular pathways. Elucidating their adaptive strategies is instrumental to understand how life can thrive in cold ecosystems and to tailor enzymes for biotechnological applications at low temperatures. In this work, we used X-ray crystallography, *in solution* studies and molecular dynamics simulations to reveal the structural basis for cold adaptation of the GH1 β-glucosidase from *Exiguobacterium antarcticum* B7. We discovered that the selective pressure of low temperatures favored mutations that redesigned the protein surface, reduced the number of salt bridges, exposed more hydrophobic regions to the solvent and gave rise to a tetrameric arrangement not found in mesophilic and thermophilic homologues. As a result, some solvent-exposed regions became more flexible in the cold-adapted tetramer, likely contributing to enhance enzymatic activity at cold environments. The tetramer stabilizes the native conformation of the enzyme, leading to a 10-fold higher activity compared to the disassembled monomers. According to phylogenetic analysis, diverse adaptive strategies to cold environments emerged in the GH1 family, being tetramerization an alternative, not a rule. These findings reveal a novel strategy for enzyme cold adaptation and provide a framework for the semi-rational engineering of β-glucosidases aiming at cold industrial processes.

To function at cold environments, psychrophilic enzymes need to overcome the reduction of chemical reaction rates induced by low temperatures^1^. This can be reached by increasing the turnover number (*k*_cat_), decreasing substrate affinity (K_m_) or changing both parameters^2^. As a result, cold-adapted enzymes are more efficient at temperatures between 0 and 30 °C than their mesophilic counterparts^3^. This attribute made them attractive biocatalysts for many applications such as to save energy in large-scale processes (dispensing the costly heating of reactors); to transform heat-sensitive products in the food industry; and to bio-remediate contaminated soils and waters in cold regions^4^.

Cold adaptation typically involves an increase in the conformational flexibility of the active site or protein surface at the expense of a decrease in the enzyme stability^1^,^4^,^5^. Several structural alterations-inside or outside the catalytic center-can improve flexibility including higher accessibility to the active site, lower content of proline and arginine residues, weaker hydrophobicity, less abundant weak interactions (electrostatic, aromatic, hydrogen bond and helix dipole), lower content of solvent exposed ion pairs and higher proportion of non-polar groups exposed to the solvent^1^,^3^,^4^. Each protein family seems to have established its own strategy to adapt to cold, using one or a combination of the aforementioned structural modifications^4^. However, for several enzymes of industrial interest, such as β-glucosidases, the molecular modifications that lead them to be cold active are still obscure.

β-glucosidases are key enzymes in cellulose depolymerization, acting synergistically with endoglucanases and cellobiohydrolases. They catalyze the breakdown of cellobiose and short cello-oligomers into glucose, the final step of cellulose saccharification. β-glucosidases with high activity at low and moderate temperatures have been of much interest for flavor enhancement in wines^6^,^7^ and production of cellulosic biofuels via simultaneous saccharification and fermentation^8^ (SSF)–a process in which cell wall-degrading enzymes operate at typical fermenting conditions of *Saccharomyces cerevisiae*. The current commercial enzyme cocktails for bioethanol production consist of meso- or thermophilic enzymes with optimum temperature around 50 °C, being not compatible with the SSF process. In comparison to other technologies at higher temperatures, SSF has several advantages including lower energy consumption, reduced undesirable chemical reactions induced by high temperatures and mitigation of product inhibition of the enzyme cascade^9^,^10^.

To handle with the abundance of substrates and glucose levels during cellulose saccharification, industrial β-glucosidases should exhibit high turnover rates and tolerance to glucose, respectively. β-glucosidases from the family GH1^11^,^12^ typically display higher tolerance to glucose than those of the family GH3, but a great challenge is to find GH1 enzymes with enhanced turnover number (*k*_cat_)^13^.

Recently, we have characterized a cold-adapted member from *Exiguobacterium antarcticum* B7 (*Ea*BglA) with higher *k*_cat_ over mesophilic counterparts at 30 °C^14^,^15^,^16^. Besides being cold-active, *Ea*BglA is also glucose tolerant, indicating that cold-adapted GH1 β-glucosidases might be instrumental to make biofuels production via SSF economically viable.

The studies with psychrophilic β-glucosidases are still scarce and limited to their isolation and biochemical characterization^17^,^18^, hampering the molecular comprehension of how nature has engineered these enzymes to work at low temperatures. Therefore, in this study, we aimed to shed light on the structural basis for cold adaptation and glucose tolerance of *Ea*BglA. For this purpose, we combined X-ray crystallography, *in solution* studies and molecular dynamics simulations to find out structural features that allowed this enzyme to adapt to the low temperatures of Antarctic lakes. The elucidation of the molecular adaptations that rendered this enzyme cold active and glucose tolerant expands our current understanding about enzyme cold adaptation and provides structural data to support the semi-rational design of high-performance β-glucosidases for biotechnological applications.

## Results

### Cold adaptation involves mutations outside the active site

**Figure 1:**
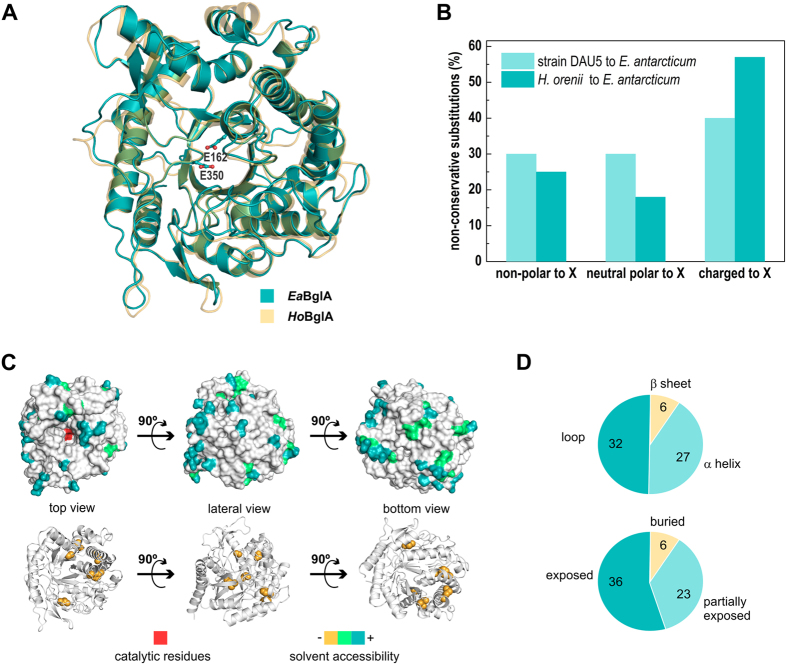
Structure and sequence analyses of the cold-adapted *Ea*BglA. (**A**) Cartoon representation of *Ea*BglA superimposed on the thermophilic *Ho*BglA, showing the catalytic residues as sticks. (**B**) Fraction of non-conservative substitutions referent to non-polar, neutral polar or charged residues from the enzymes DAU5-BglA and *Ho*BglA replaced by residues of a different category (X) in *Ea*BglA. (**C**) Residues specific to the cold-adapted enzyme (according to a sequence alignment between *Ea*BglA, DAU5-BglA and *Ho*BglA) mapped in the 3D structure and colored according to its solvent accessibility from yellow (buried) to blue (fully exposed). Catalytic glutamates are shown in red. (**D**) Number of residues specific to the cold-adapted enzyme classified according to their localization in the protein secondary structure (top chart) and solvent accessibility (bottom chart).

Crystallographic studies showed that the (α/β)_8_-barrel fold and the catalytic residues Glu^162^ (acid/base) and Glu^350^ (nucleophile), typical of GH1 members^19^,^20^, are conserved in *Ea*BglA (Fig. 1A). Interestingly, the cold-adapted *Ea*BglA is closely related to a thermostable β-glucosidase from *Halothermotrix orenii* (*Ho*BglA)^21^ (Fig. 1A), sharing 48% of sequence identity that, as expected, reflects in a high structural similarity with only small main-chain displacements in peripheral loops and α-helices (Table 1, Fig. 1A).

To identify the molecular adaptations that lead EaBglA to be cold active, we compared the structure and sequence of *Ea*BglA with two β-glucosidases with progressively lower sequence identity and higher optimal temperatures: the BglA from *Exiguobacterium* sp. DAU5 strain (83%; 45 °C)^22^ and *Ho*BglA (48%; 65–70 °C)^21^ (Table 1, Supplementary Fig. S1). The three proteins have identical catalytic centers (-1 subsite), suggesting that cold adaptation resulted from modifications in other regions (Supplementary Fig. S1A).

In order to understand the changes involved in cold adaptation, we analyzed the 15% out of the 435 aligned residues that are specific to the cold-adapted enzyme (Supplementary Fig. S1B). Most of them refer to non-conservative substitutions, mainly of charged residues in the meso/thermophilic enzymes for neutral residues in *Ea*BglA (Fig. 1B). This trend correlates with a lower content of charged residues with long side chains (Arg, Glu and Lys) and a higher ratio of non-polar and polar residues in *Ea*BglA compared to the thermo/mesophilic proteins (Table 1). Few residues were replaced at the protein core, preserving the number of intramolecular hydrophobic interactions (21 at *Ea*BglA and 20 at *Ho*BglA). These structural analyses showed that cold-specific substitutions are dispersed mainly at the solvent-exposed surface of loops and α-helices (Fig. 1C,D), indicating that the redesign of the protein surface contributed to the cold adaptation of *Ea*BglA.

### The cold-active EaBglA adopts a novel tetrameric arrangement among GH1 enzymes

**Figure 2:**
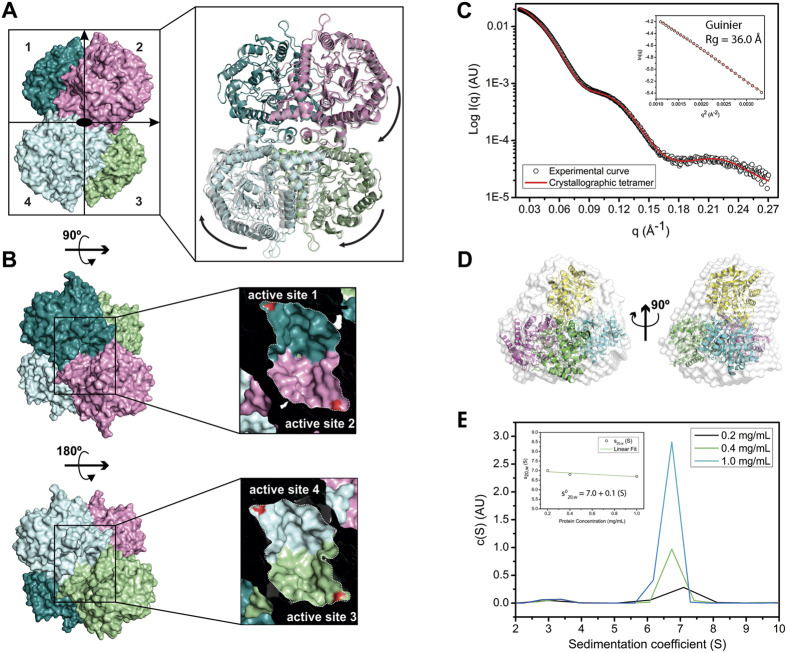
The unique tetrameric structure of *Ea*BglA. (**A**) The crystal structures of *Ea*BglA reveal a tetrameric arrangement with 222 symmetry. The two independent tetramers observed in the *P*2_1_ and *C222*_*1*_ crystals (multicolor and gray) are in slightly different conformations, suggesting inter-subunits motions (arrows). (**B**) Two pairs of interfacing active sites (1–2 and 3–4) locate at opposite faces of the tetramer. SAXS profile (**C**) and *ab-initio* model (**D**) agree with the theoretical curve and high-resolution structure of the tetramer. (**E**) AUC assays show the presence of tetramers in solution, even at low protein concentrations.

The two crystallographic structures of *Ea*BglA, solved in distinct space groups, presented the same tetrameric arrangement (Fig. 2A). Structural comparisons revealed that the *Ea*BglA subunits assemble in a different way from those of GH1 tetramers available at the Protein Data Bank (PDB), indicating that the tetrameric arrangement of *Ea*BglA is the first of its kind to be reported within the GH1 family (Supplementary Fig. S2).

Hydrodynamic studies using SAXS and AUC indicate that the *Ea*BglA tetramer is stable in solution (Fig. 2C–E, Supplementary Table S1). The SAXS scattering curve displays excellent agreement with the theoretical curve of the crystallographic tetramer (Fig. 2C) and a low spatial discrepancy is observed between the crystal structure and the low-resolution SAXS envelope (Fig. 2D). Moreover, AUC assays detected tetramers in solution even at a protein concentration 10-fold lower than that used to collect SAXS data (Fig. 2E).

**Figure 3:**
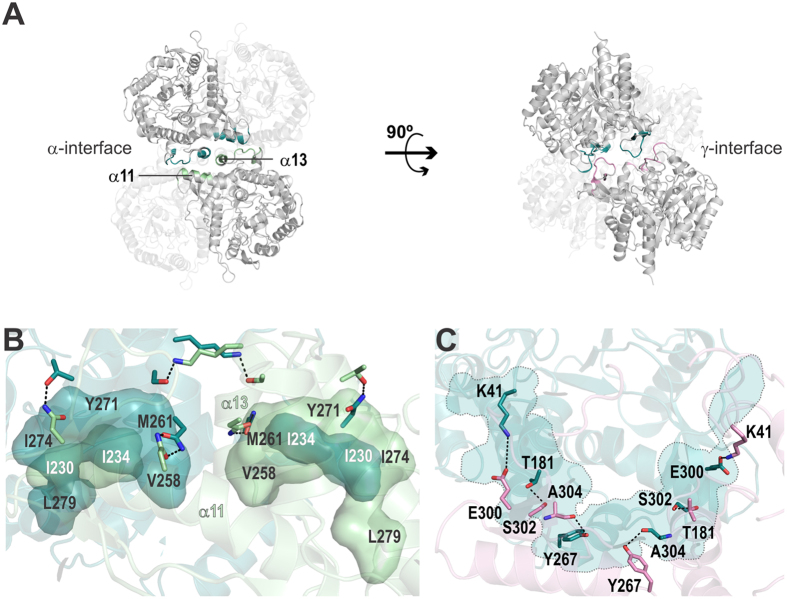
The interfaces of *Ea*BglA tetramer. (**A**) The interface α involves residues from the α-helices α11 and α13, while the interface γ connects loops from the catalytic face. (**B**) Two hydrophobic clusters (*labeled residues*) with a two-fold symmetry stabilize the interface α along with few hydrogen bonds (*dotted lines*) between interfacing polar residues (*sticks*). (**C**) The interface γ is essentially linked by hydrogen bonds (*dotted lines*) and electrostatic interactions (Lys^41^-Glu^300^), forming a semi-circle (*light blue*) that connects two active sites.

The tetramer has a 222 symmetry (with a distorted tetrahedral geometry) and two pairs of active sites placed at opposite faces (Fig. 2A,B). Each pair shares the same entrance and forms a huge cavity [11393 Å^3^ ± 216 (mean ± SD)] with a two-fold symmetry (Fig. 2B). Two types of interface stabilize the *Ea*BglA tetramer (called here α and γ). The type α is a side-to-side interface composed by residues from the helices α11 and α13 and the loops α13/α14 and β8/β9 (Fig. 3A). It comprises two hydrophobic clusters surrounded by few hydrogen bonds (Fig. 3B). The γ interface forms a semi-circle wall at the catalytic face of the TIM barrel, connecting the active sites from neighboring subunits (Fig. 3C,D). It comprises polar residues involved in the formation of several hydrogen bonds (Fig. 3D).

*Ea*BglA crystallized in slightly different conformations, as indicated by the structural superimposition of the independent tetramers observed in the *P*2_1_ and *C*222_1_ crystalline forms (Fig. 2A). These conformational changes result in variations in the surface area buried into the tetramer interfaces and in the volume of the active sites, suggesting that *Ea*BglA forms a flexible oligomer, which possibly contribute to overcome the structural rigidity imposed by low temperatures (Supplementary Table S2).

### The tetramer is the biologically active unit of EaBglA

**Figure 4:**
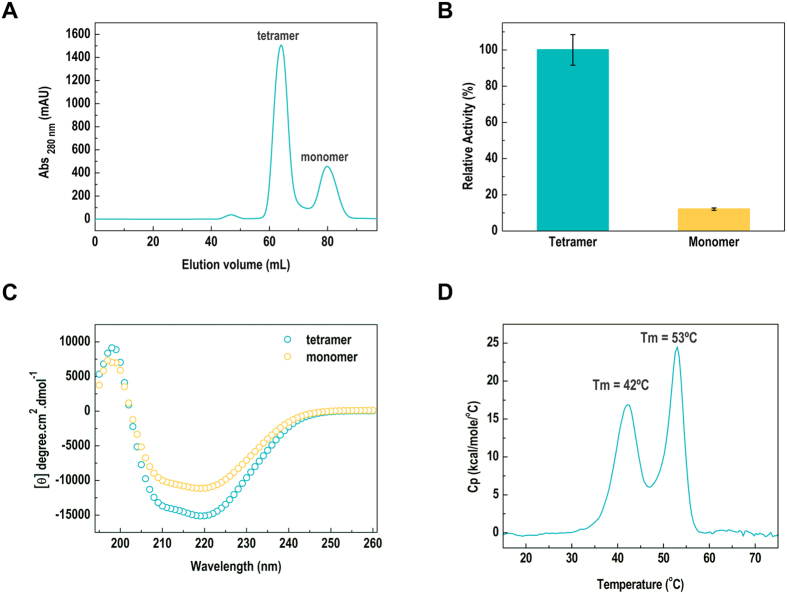
The tetramer is the active form of *Ea*BglA. (**A**) The SEC profile of *Ea*BglA displays two peaks. The former correspond to tetramers, as determined by SAXS, while the second refers to a monomeric population according to DLS analyses (R_h_ = 3.8 ± 1.2 nm; polydispersity = 30%; estimated mass ~87 ± 26 kDa). (**B**) The monomers are about 10-fold less active than tetramers, indicating that the quaternary structure is crucial for the proper enzyme function. (**C**) CD spectra show conformational differences between monomers and tetramers at the secondary structure level. (**D**) DSC assays reveal that *Ea*BglA presents two distinct calorimetric units, possibly representing tetramer dissociation (at 42 °C) prior to protomer unfolding (at 53 °C).

Using size-exclusion chromatography (SEC), we isolated a small fraction of monomers from the predominant population of tetramers, as confirmed by DLS analysis (Fig. 4A). The monomer is about ten-fold less active than the tetramer, indicating that the quaternary structure is crucial for the proper function of the enzyme (Fig. 4B). Circular dichroism assays also showed differences at the secondary structure level between monomers and tetramers, supporting that the oligomeric assembly stabilizes the native conformation of the enzyme (Fig. 4C).

The stability of *Ea*BglA tetramer was examined by differential scanning calorimetry (DSC) and the denaturation curve showed two well-defined transitions, indicating the presence of two calorimetric units (Fig. 4D). Since two populations of *Ea*BglA–tetramers and monomers–were detected by size-exclusion chromatography, the first and second thermal transitions might reflect the dissociation of the tetramer into folded monomers followed by monomer denaturation.

### Tetramerization increases flexibility at the protein surface

**Figure 5:**
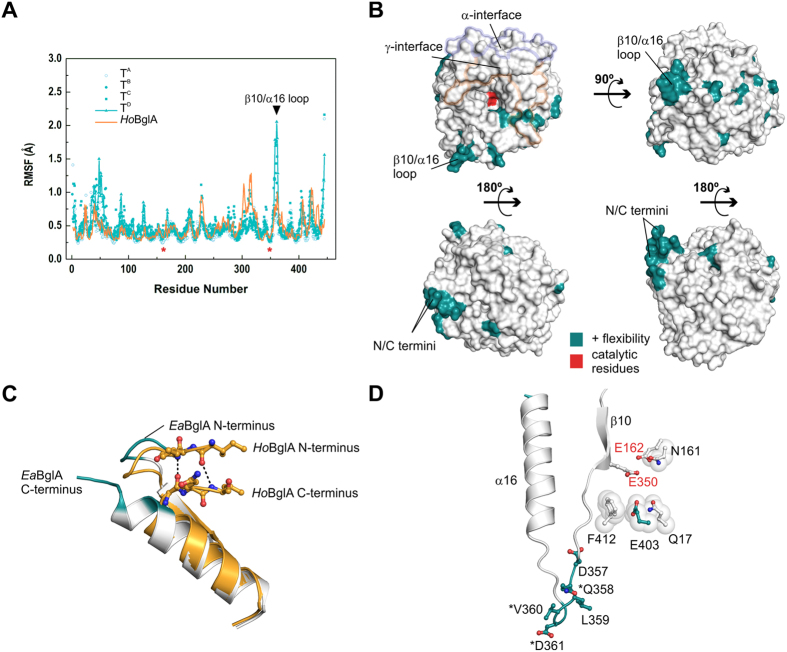
The tetramer promotes local changes in flexibility. (**A**) Comparison of the backbone root mean square fluctuations (RMSF) of *Ea*BglA tetramer chains (T^A^-T^D^) and the *Ho*BglA monomer at 10 °C. For comparison purposes, the RMSF profiles were aligned according to the structural alignment between *Ea*BglA and *Ho*BglA (assuming *Ea*BglA numbering as reference). The position of the catalytic residues (asterisk) and the β10/α16 loop (triangle) are indicated. (**B**) Surface representation of an *Ea*BglA subunit highlighting the catalytic residues (red), the tetramer interfaces (α and γ) and the residues with higher flexibility (dark cyan) compared to *Ho*BglA (ΔRMSF > 0.25 Å in at least two tetramer subunits). Protein views correspond to Fig. 1C. N- and C-termini as well as the β10/α16 loop are indicated. (**C**) N- and C-termini of *Ho*BglA (orange) superimposed onto *Ea*BglA (colored according to panel B). Dashed lines represent main-chain hydrogen bonds connecting N- and C-terminal residues (sticks) in the thermophilic enzyme. (**D**) The solvent-exposed residues of β10/α16 loop (dark cyan sticks) are more flexible in the cold-adapted enzyme. Some of them are specific to *Ea*BglA compared to *Ho*BglA and Dau5-BglA (asterisk). The β10/α16 loop are near Phe^412^. The latter along with Glu^403^, Gln^17^ and Asn^161^ delineate the -1 subsite and display higher RMSF values than their *Ho*BglA counterparts do in most tetramer subunits, according to the MD simulation at 10 °C. Red labels indicate catalytic residues.

To gather insight into the dynamic properties of the tetramer and its potential role in cold adaptation, we performed molecular dynamics simulations using the biological units of *Ea*BglA (tetramer) and *Ho*BglA (monomer)^21^. The two proteins have similar overall flexibility, but with local changes that might affect enzymatic activity (Table 2, Fig. 5A).

The local changes in the flexibility of *Ea*BglA in relation to the thermophilic *Ho*BglA correlate with a lower content of salt bridges and a higher exposure of hydrophobic regions to the solvent in the cold-adapted enzyme (Table 2). Although the total number of hydrogen bonds is lower in *Ea*BglA, the average value of H bonds per simulation frame is virtually identical in both enzymes, suggesting that the size of their intramolecular networks was conserved even under distinct selective pressures.

The regions with the highest gains in flexibility-compared to the thermophilic enzyme-locate at the *Ea*BglA surface and include the N- and C-termini as well as the solvent-exposed region of the β10/α16 loop (Fig. 5A,B). The higher motility at the N- and C-termini of *Ea*BglA is probably due to a C-terminal insertion that disrupts a molecular zipper that links the N and C extremities of the thermophilic enzyme (Fig. 5C, Supplementary Fig. S1B). The β10/α16 loop harbors cold-specific mutations and is adjacent to the active-site residue Phe^412^ (Fig. 5D). This residue, along with Glu^403^, Gln^17^ and Asn^161^, displays higher motility in most tetramer subunits, compared to its counterpart in the *Ho*BglA active site. The proximity between the β10/α16 loop and Phe^412^, Glu^403^ and Gln^17^ suggests that the enhanced surface entropy of the β10/α16 loop is propagated to the active site and likely affects the catalytic rates at low temperatures.

### Residues related to glucose tolerance are conserved in *Ea*BglA

**Figure 6:**
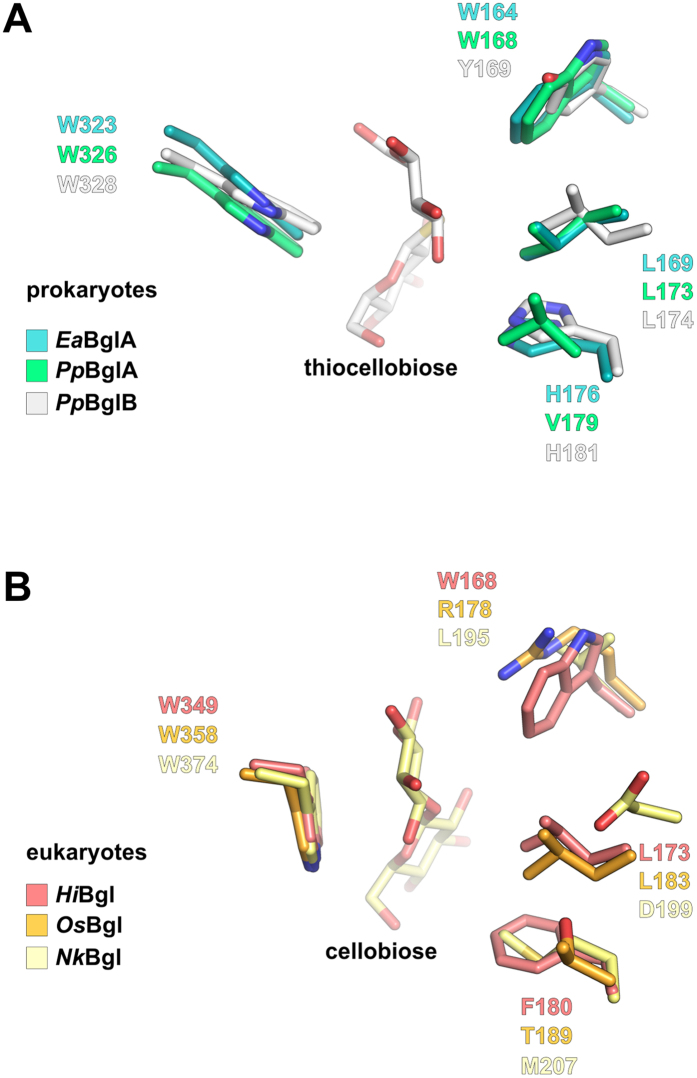
Residues involved in glucose tolerance are conserved in the aglycone-binding site of *Ea*BglA. (**A**) *Ea*BglA superimposed on the *Pp*BglA (PDB ID: 1E4I) and *Pp*BglB-complexed with thiocellobiose–(PDB ID: 2O9R) from *P. polymyxa.* (**B**) Structural alignment between three eukaryotic β-glucosidases: the fungal *Hi*Bgl (PDB ID: 4MDP)^12^, the plant *Os*Bgl (PDB ID: 2RGL)^15^ and the insect *Nk*Bgl (complexed with cellobiose; PDB ID: 3VIK)^59^. The highly conserved residue Trp^323^ has an orientation exclusively found in bacterial enzymes (**A**) that induces a different conformation of the reducing-end sugar compared to eukaryotic enzymes (**B**). The residues Trp^164^ and Leu^179^, suggested to play a role in glucose tolerance in HiBgl (**B**) and other GH1 enzymes^12^,^24^, are also conserved in *Ea*BglA (**A**).

Besides being a cold-active enzyme, *Ea*BglA is also glucose tolerant^14^; a desirable feature in bioprocesses with high production of glucose, such as biomass saccharification^23^. Interestingly, the residues Trp^164^, Leu^169^ and His^176^, considered to play a role in the glucose tolerance of some GH1 β-glucosidases^12^,^24^, are conserved in *Ea*BglA. The amino acid triad X-Leu-His (X for aromatic residues) is also conserved in the more glucose-tolerant BglB from *Paenibacillus polymyxa*^25^, while the less glucose-tolerant BglA from the same bacterium^26^ has a valine replacing His^176^, supporting the influence of this residue in glucose tolerance (Fig. 6A).

Structural comparisons between *Ea*BglA and a closely related homologue in complex with thiocellobiose^25^ showed that Trp^164^ and Leu^169^, along with His^176^ and Trp^323^, delineate a hydrophobic microenvironment that accommodates and orientates the reducing end portion of the substrate (Fig. 6A). Apart from Trp^323^, the other residues composing the aglycone-binding site are variable among β-glucosidases, contributing to distinct kinetic properties and substrate specificity^27^. As expected, the highly conserved Trp^323^ assumes an orientation similar to that found in other bacterial β-glucosidases, but it diverges from that observed in eukaryotic enzymes (Fig. 6). Changes in the Trp^323^ orientation seem to be unrelated to glucose tolerance, since some prokaryotic and eukaryotic enzymes can tolerate similar levels of this monosaccharide^28^.

### Cold adaptation in the GH1 family seems to have emerged from distinct molecular routes

**Figure 7:**
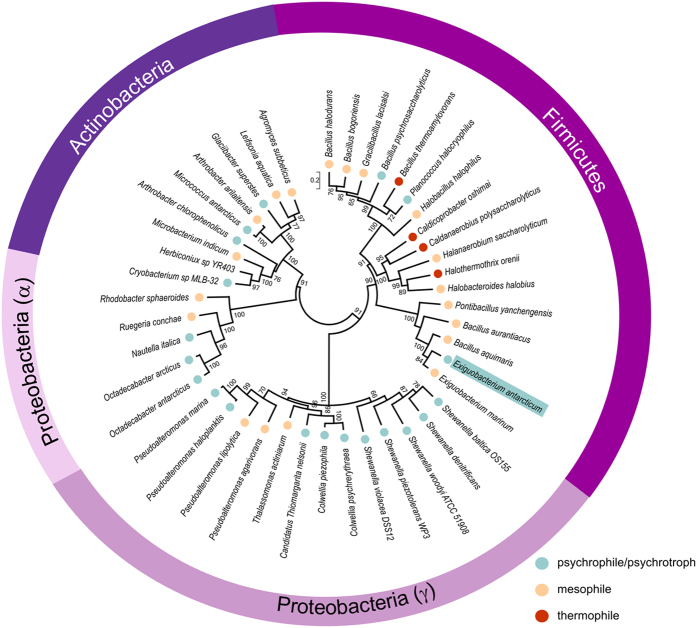
Phylogenetic tree of GH1 β-glucosidases in bacteria. Unrooted tree of 44 protein sequences of GH1 β-glucosidases as inferred by the ML method using the MEGA6^57^ program. Numbers at nodes represent bootstrap values from 1000 replications (shown as percentage and only those higher than 50%). Scale bar indicates estimated number of substitutions per site. Taxonomic classification at the phylum level (Actinobacteria, Firmicutes and Proteobacteria) are indicated, subdividing the Proteobacteria phylum into the two classes represented in the tree (α and γ). Colored circles indicate the classification of each specie as psychrophile/psychrotroph (*blue*), mesophile (*light orange*), and thermophile (*red*) according to literature data (see Table S5). Cyan box indicates the *E. antarcticum* position in the tree.

To evaluate if the strategy that rendered *Ea*BglA cold active is conserved in the GH1 family, we constructed a phylogenetic tree based on protein sequences of GH1 β-glucosidases from psychrotrophic/psychrophilic bacteria and their closest relatives (Fig. 7). This analysis revealed that cold-adapted β-glucosidases have emerged several times during the evolution of bacteria and in different phyla such as Firmicutes, Actinobacteria and Proteobacteria (Fig. 7). Therefore, the set of molecular modifications selected by nature to produce cold-active β-glucosidases might be different in these distantly related enzymes. Supporting this hypothesis, multiple sequence alignment shows that the residues forming the tetrameric interfaces of *Ea*BglA are poorly conserved in other enzymes from psychro-tolerant bacteria, suggesting that this particular oligomerization strategy emerged specifically in the *Exiguobacterium* clade (Supplementary Fig. S3).

## Discussion

In this work, we found that the molecular strategies that lead *Ea*BglA to be cold-active targeted superficial residues from peripheral helices and loops, with a trend to replace charged by neutral amino acids. Compared to thermo-tolerant enzymes, *Ea*BglA displays a higher content of residues that favor flexibility including alanine, which makes few side chain interactions, and threonine, which is prone to form hydrogen bonds with water^29^. Similarly to other cold-adapted enzymes, *Ea*BglA has a lower content of arginine and other charged residues compared to the thermophilic *Ho*BglA, reflected by almost two-fold less salt bridges and a higher exposure of hydrophobic areas to the solvent in the cold-active enzyme.

As expected, *Ea*BglA has a lower thermo-tolerance compared to the thermophilic *Ho*BglA^21^. Our analyses suggest that the lower thermo-tolerance of *Ea*BglA is due to changes in superficial loops and to a C-terminal insertion, which disrupts a molecular zipper between the N and C-terminal regions. Random mutagenesis studies in a homologue protein from *P. polymyxa* further support that solvent-exposed residues located in loops play a role in β-glucosidase thermo-resistance^30^.

The redesign of *Ea*BglA surface originated a tetrameric configuration, a rare strategy among psychrotolerant proteins, which generally are monomeric or form lower-order oligomers than their mesophilic relatives^29^. Our data also revealed that the tetramer stabilizes the native conformation of the enzyme, leading to a 10-fold higher activity compared to monomers.

*Ea*BglA thermal inactivation^14^ coincides with its first calorimetric transition, which probably represents tetramer disassembly into partially-folded and poorly active monomers. The maximum enzyme activity observed for the tetramer at 30 °C decreases to 15% at 45 °C^14^, an intermediate temperature between the first and second calorimetric units, which correlates with the monomer retaining about 10% of tetramer activity.

According to CD and AUC analyses, the monomeric fraction isolated by SEC might consist of a subpopulation of molecular specimens with improper conformation that is unable to tetramerize. Comparison of CD spectra of monomers and tetramers indicate conformational changes that could result from the local unfolding of α-helices and β-sheets in the monomers (Fig. 4C). Moreover, tetramer dissociation was not observed upon protein dilution from 1 to 0.2 mg/mL (Fig. 2E), suggesting that the tetramers and monomers isolated by SEC (samples at 4 mg/mL) do not reflect a binding equilibrium under the tested conditions. In addition, the monomeric fraction showed to be unstable, precipitating after short-time storage, which corroborates with an improper conformation of monomers.

The *Ea*BglA tetramer has increased flexibility mainly at surface exposed patches placed at the catalytic face. The local softening of the protein surface probably contribute to reduce the enthalpy of activation of the catalyzed reaction at the expense of a more negative entropic component (-TΔS), as commonly observed in cold-adapted enzymes^5^,^31^,^32^,^33^. This strategy makes the reaction rates less sensitive to temperature and possibly counterbalance the structural rigidity imposed by cold conditions, favoring catalytic activity^5^.

Although most GH1 enzymes of known structure are monomeric, tetramerization seems to be a tendency among GH1 enzymes from hyperthermophilic archaea (Supplementary Tables S3–S4). Structural comparisons between *Ea*BglA and these tetramers showed unrelated interfaces, supporting they have distinct evolutionary origins (Supplementary Fig. S2). Thus, we may conclude that tetramerization was employed either as a strategy to adapt GH1 enzymes to low or to extremely high temperatures, but using distinct molecular routes in response to different selective conditions.

Cold adaptation emerged several times during the evolution of bacterial GH1 β-glucosidases, but not necessarily was achieved using tetramerization as a strategy. For example, the β-glucosidase from *Micrococcus antarcticus* (*Ma*Bgl) and the protein BglMKg from a close relative of *Shewanella* spp. are monomeric^17^,^18^. *Ma*Bgl has a wide insertion in the β8/β9 loop that seems to prevent the formation of the γ-interface (Supplementary Fig. S3C). Moreover, the residues from the α and γ interfaces of *Ea*BglA tetramer are poorly conserved in other cold-adapted GH1 β-glucosidases, suggesting they do not share the same oligomeric state (Supplementary Fig. S3A).

The idea that multiple solutions for cold adaptation emerged during the evolution of the GH1 family is likely applicable to other enzyme families and supported by directed evolution studies, which can identify adaptive mechanisms different from those found in nature^34^. As only two GH1 β-glucosidases from psychrotrophic/ psychrophilic species have known structures so far, further structural studies are required to better understand how nature has created cold-active GH1 members by redesigning the same scaffold in different ways.

Exploring this natural variability via semi-rational protein engineering approaches might be useful to tailor enhanced enzymes for biotechnological applications in which low temperatures are required. The ethanol production via simultaneous saccharification and fermentation, for example, could benefit of cold-active β-glucosidases. Firstly, because this process requires a compromise between the optimal temperature for enzymatic hydrolysis and that for fermentation by *S. cerevisae*, which is near 30 °C^35^. Secondly, because enzymes with increased *k*_cat_ at low temperatures-which is likely the case of cold-adapted β-glucosidases over the commercial meso/thermophilic enzymes-would perform better at the high concentrations of cellobiose generated during saccharification.

## Materials and Methods

### Protein overexpression and purification

The *Ea*BglA-pET28a expression vector was transformed into *Escherichia coli* BL21(DE3) cells (Agilent Technologies, Santa Clara, USA). The cells were grown in 1L of LB medium at 37 °C and protein expression was induced with 0.4 mM isopropyl -D-thiogalactopyranoside (IPTG) at 37 °C for 3 h. The cells were harvested by centrifugation at 7,000 *xg* (20 min, 4 °C), resuspended in buffer A (20 mM sodium phosphate, 500 mM NaCl, 20 mM imidazole, pH 7.5), supplemented with 1 mM PMSF, and lysed by lysozyme treatment followed by sonication. The cell extract was clarified by centrifugation (20,000 *xg*, 30 min, 4 °C) and the supernatant was applied onto 5 mL Hi-Trap chelating HP column (GE Healthcare Biosciences, Pittsburgh, USA) coupled to an ÄKTA system (GE Healthcare Biosciences, Pittsburgh, USA) and pre-equilibrated with buffer A. The bound proteins were eluted with a nonlinear gradient of buffer B (20 mM sodium phosphate, 500 mM NaCl, 500 mM imidazole, pH 7.5). The fractions with β-glucosidase activity were pooled, concentrated to 5 mL using Amicon Ultra centrifugal units and submitted to size-exclusion chromatography using a Superdex 75 16/60 column (GE Healthcare Biosciences, Pittsburgh, USA) previously equilibrated with 20 mM sodium phosphate, 150 mM NaCl, pH 7.5, at a flow rate of 0.5 mL/min. The tetrameric and monomeric forms were successfully separated in two different peaks by this chromatographic step and showed high purity according to SDS-PAGE analysis. Protein concentration was estimated by A_280nm_ using the extinction coefficient of the 110,030 M^−1^cm^−1^.

### Biochemical enzyme assays

Enzyme activity was measured using the colorimetric substrate 4-nitrophenyl β-D-glucopyranoside (*p*NPG, Sigma-Aldrich Co, St. Louis, USA). Experiments were carried out in triplicate in 100 μL reactions at substrate saturating conditions (0.5 mM *p*NPG) in 40 mM sodium phosphate buffer pH 7.0. The final enzyme concentration was 20 nM. The reactions were incubated for 10 min at optimum conditions for catalytic activity (30 °C and pH 7.0) and stopped with 100 μL of 1 M sodium carbonate (Na_2_CO_3_). The enzyme activity was measured spectrophotometrically at 405 nm monitoring the release of *p*-initrophenol using an Infinite® 200 PRO microplate reader (TECAN Group Ltd., Männedorf, Switzerland). The measurements were expressed as relative activity (%) considering the maximum catalytic activity observed for the biological unit of the enzyme (tetramer).

### Circular Dichroism

Circular Dichroism spectroscopy was employed to assess the global conformation of *Ea*BglA in both tetrameric and monomeric conformations. CD measurements were acquired on a JASCO J-815 CD spectrometer controlled by a CDF-426S/15 Peltier temperature control system (Jasco Analytical Instruments, Oklahoma, EUA). A quartz cuvette with a 1-cm path length was used for all CD experiments and each spectrum was an average of at least three scans. Protein concentration was 5.8 μM (for tetramer and monomer) in 20 mM sodium phosphate, 150 mM NaCl, pH 7.5. All spectra were obtained at 20 °C in the range 200–260 nm with a bandwidth of 2 nm and a response time of 4 s/nm. CD data were buffer subtracted and normalized to molar residual ellipticity allowing the comparison between the forms.

### Dynamic light scattering

Hydrodynamic radius (R_h_) of the monomeric form of *Ea*BglA was determined by Dynamic Light Scattering (DLS). Data were recorded at 20 °C on a Malvern Zetasizer Nano ZS 90 (Malvern Instruments, Worcestershire, UK) with a 633 nm laser, in a quartz cell with a scattering angle of 90°. The samples purified from the size-exclusion chromatography were analyzed in different concentrations (0.3 to 1.2 mg/mL) and the hydrodynamic radii were obtained after the average of 20 runs from the extrapolation of the translational diffusion coefficient (Dt) according to the Stokes–Einstein equation.

### Analytical ultracentrifugation (AUC)

Sedimentation velocity (SV) experiments were conducted using a Beckman Optima XL-A analytical ultracentrifuge (Beckman Coulter Inc, Brea, EUA). Data were collected at 35,000 rpm and 20 °C using the absorbance optical system for both 220 and 280 nm detections. *Ea*BglA was prepared in different concentrations (0.2, 0.4 and 1 mg/mL) in 20 mM sodium phosphate, 150 mM NaCl, pH 7.4. The SV scans were analyzed using the c(s) method in the SEDFIT program (v14.4d)^36^ in order to determine the molecular mass of the protein. A conventional c(s) distribution was applied with a fixed regularization confidence level of 0.95 and the frictional ratio (ƒ/ƒ_0_) used as a regularization parameter. The standard sedimentation coefficients (s_20,w_) were determined by the maximum of the peaks of the continuous c(S) curves after corrections to eliminate the interferences caused by the buffer viscosity, density and temperature (ρ = 1.0039 g/mL and η = 0.0102643 Poise were obtained by the SEDNTERP program). The s°_20,w_ value at infinite dilution was obtained by the linear regression of s_20,w_ as a function of protein concentration^37^.

### Small angle X-ray scattering

Small angle X-ray scattering (SAXS) measurements were performed using a monochromatic X-ray beam (λ = 1.488 Å) from the D01A-SAXS2 beamline at the Brazilian Synchrotron Light Laboratory (LNLS, Campinas, Brazil). *Ea*BglA samples were prepared in 20 mM sodium phosphate, 150 mM NaCl, pH 7.4 at the concentrations of 1 and 2 mg/mL. The samples were centrifuged for 30 min at 23,000 *xg* and 4 °C to remove potential aggregates before all SAXS experiments. The sample-to-detector distance was set to 1000 mm to obtain the range of the scattering vector (q) from 0.013 to 0.33 Å^−1^, where q means the magnitude of the q-vector defined by q = 4π sinθ/λ (2θ is the scattering angle). The samples were analyzed at 20 °C in 1-mm path-length mica cells and the scattering profiles were recorded in ten successive frames (each of 10 s duration) to monitor radiation damage and beam stability. The obtained SAXS curves were buffer subtracted and integrated using the FIT2D program^38^. The radius of gyration (R_g_) was determined from the Guinier equation^39^ and by indirect Fourier transform method using the GNOM package^40^. The GNOM program was also used to generate the particle distance distribution p(r) and the maximum diameter, D_max_. The DAMMIN program^41^ was applied to obtain *ab initio* models for *Ea*BglA (dummy atom model), by a simulated annealing optimization routine that best fit to the experimental scattering data. The protein shape was reconstructed by averaging 20 different *ab initio* models using the DAMAVER package^42^. The obtained low-resolution model and the crystal structure were superimposed using the SUPCOMB program^43^. The theoretical scattering curve was generated from crystallographic atomic coordinates using the CRYSOL program^44^ and compared to the experimental scattering data.

### Differential scanning calorimetry

Thermal stability of *Ea*BglA was investigated by differential scanning calorimetry (DSC). *Ea*BglA, at a concentration of 2 mg/mL in 20 mM sodium phosphate, 150 mM NaCl and pH 7.4, was scanned at a rate of 1 °C/min in a VP-DSC device (Microcal, GE Healthcare, Northampton, USA) over the range of 15–90 °C at 0.5 °C increments. The DSC profile was buffer subtracted, concentration normalized and the resultant endotherms integrated following assignment of pre- and post-transition baselines. As the thermal-induced unfolding of *Ea*BglA was irreversible in the tested conditions, the analysis was focused on the values of transition temperatures (T_m_).

### Protein crystallization

Crystallization experiments were performed by the vapor diffusion method in 96-well plates using a Honeybee 963 automated system (Digilab, Marlborough, MA). *Ea*BglA crystallized at 18 °C in sitting drops containing 0.5 μl of protein solution at 15 mg/mL and 0.5 μL of the crystallization condition. *C*222_1_ crystals were grown in a condition containing 0.1 M CAPS (pH 10.5), 0.2 M lithium sulfate and 2 M ammonium sulfate using *in situ* proteolysis (1:1000 *(w/w)* ratio of trypsin:*Ea*BglA). *P*2_1_ crystals were obtained in a medium containing 0.1 M TRIS (pH 8.5), 2% *(v/v)* PEG400 and 1.45 M lithium sulfate.

### Data collection and structure determination

X-ray diffraction data of *C*222_1_ and *P*2_1_ crystals (cryoprotected with 20% *(v/v)* glycerol) were collected at the MX2 beamline from LNLS (Campinas, Brazil) and at the I03 beamline from the Diamond Light Source (Oxfordshire, UK), equipped with PILATUS 2M and PILATUS3 6M detectors, respectively. Data were indexed, integrated and scaled using the XDS package^45^. The crystal structure was solved by molecular replacement methods using the PHASER program with the atomic coordinates of the β-glucosidase from *H. orenii* (PDB ID: 4PTV) as a search model. The structures were refined alternating cycles of TLS, twin refinement (*P*2_1_ crystal) and restrained refinement (including local NCS restraints) using REFMAC5^46^ and manual model building using COOT^47^. The two last residues were disordered in all chains from both crystals and were not modeled. The final structures were validated using MOLPROBITY^48^. Data collection and refinement statistics are summarized in Table 3. The structure factors and atomic coordinates of both crystalline forms, P2_1_ and C222_1_, were deposited in the PDB under the accession codes 5DT5 and 5DT7, respectively.

### Structural analyses

Structural superimpositions were performed using the protein structure comparison service PDBeFold (http://www.ebi.ac.uk/msd-srv/ssm)^49^. Intramolecular interactions and protein interfaces were analyzed using the servers PIC (http://pic.mbu.iisc.ernet.in/)^50^ and EPPIC (http://www.eppic-web.org/ewui/)^51^, respectively. The volume of protein cavities were calculated using KVFinder^52^ and CASTp^53^. Figures were prepared using PYMOL^54^.

### Molecular dynamics simulations

Simulation systems using explicit solvation were created for the biological units of *Ea*BglA and *Ho*BglA (PDB ID: 4PTX). A molecular dynamics simulation of 100 nanoseconds was carried out using the YAMBER3 force field with timestep of 5 femtoseconds saving a snapshot every 250 picoseconds using YASARA^55^. Each of the systems were simulated in 10 and 30 °C using the Berendsen thermostat for temperature control. Pressure was monitored using the solvent density as a probe and maintained constant during simulation by isotropically resizing the simulation cell to match adequate values of solvent density, when required.

### Molecular dynamics analyses

Root mean square fluctuation as well as hydrogen bonds, salt bridges and solvent accessible area were calculated using custom scripts and the coordinates of the last 80 nanoseconds of the 100 nanoseconds simulations. Hydrogen bonds were identified considering the bond energy of 6.25 kJ/mol as a threshold, given that the bond energy is a function of the hydrogen-acceptor distance. Electrostatic pairs were considered when their atoms (not hydrogen) were closer than 4 Å from each other. The solvent accessible surface was calculated using routines implemented in the YASARA software.

### Phylogenetic analysis

The protein sequences of β-glucosidases from psychrotrophic bacteria were used as baits in BLASTp searches against the NCBI non-redundant database. A maximum of four protein hits with a sequence identity ranging from 60 to 95% to each query were selected. For comparison purposes, homolog proteins of *Ho*BglA were also retrieved using the same criteria. All protein sequences were aligned using the MUSCLE^56^ software. Positions containing gaps or missing data were partially excluded from the multiple sequence alignment using a site coverage cutoff of 80%. Based on the resulting data subset, an evolutionary tree was inferred using the Maximum Likelihood (ML) method and the best model of amino acid substitution indicated by the MEGA software (version 6.0)^57^, which was the LG matrix assuming that the rate varies among sites according to a Gamma distribution (+G) and allowing for the presence of invariant sites (+I). The confidence of tree topology was assessed using the Bootstrap analysis based on 1,000 bootstrap replications. The taxonomy of bacteria species was retrieved from the Uniprot Knowledgebase^58^. Bacteria were classified based on their optimal growth temperature, when available, or in the minimal temperature they are able to growth as psychrophilic/psychrotrophic (T_opt_ < 20 °C; T_min_ < 5 °C), mesophilic (20 °C < T_opt_ > 40 °C; T_min_ > 5 °C) and thermophilic (T_opt_ > 40 °C; T_min_ > 40 °C) (Table S5).

## Additional Information

**How to cite this article**: Zanphorlin, L. M. *et al.* Oligomerization as a strategy for cold adaptation: Structure and dynamics of the GH1 β-glucosidase from *Exiguobacterium antarcticum* B7. *Sci. Rep.* **6**, 23776; doi: 10.1038/srep23776 (2016).

## Supplementary Material

Supplementary Information

## Figures and Tables

**Table 1:** Sequence information and structural alignment of the β-glucosidases from *H. orenii*, *E.* strain DAU5 and *E. antarcticum* B7.

Enzyme source	*H. orenii*	*E.* DAU5	*E. antarcticum*
Sequence properties			
UNIPROT code	B8CYA8	E0X9H4^a^	K0A8J9
Sequence identity to *Ea*BglA	48%	83%	
Number of residues	451	450	448
Gly number (ratio)	37 (8.2%)	31 (6.9%)	31 (6.9%)
Ala number (ratio)	30 (6.7%)	36 (8.0%)	38 (8.5%)
Pro number (ratio)	19 (4.2%)	14 (3.1%)	17 (3.8%)
Phe number (ratio)	23 (5.1%)	27 (6.0%)	30 (6.7%)
Tyr number (ratio)	27 (6.0%)	24 (5.3%)	22 (4.9%)
Ser number (ratio)	18 (4.0%)	21 (4.7%)	22 (4.9%)
Thr number (ratio)	18 (4.0%)	16 (3.6%)	22 (4.9%)
Gln number (ratio)	10 (2.2%)	8 (1.8%)	15 (3.3%)
Asp number (ratio)	36 (8.0%)	42 (9.3%)	42 (9.4%)
Glu number (ratio)	35 (7.8%)	32 (7.1%)	24 (5.4%)
His number (ratio)	15 (3.3%)	20 (4.4%)	21(4.7%)
Lys number (ratio)	28 (6.2%)	21 (4.7%)	20 (4.5%)
Arg number (ratio)	22 (4.9%)	22 (4.9%)	16 (3.6%)
Multiple 3D alignment			
PDB ID	4PTX	*homology model*^b^	5DT5
Number of residues	445	440	444
Number of aligned residues	435	435	435
R.M.S.D. *E.* DAU5 (Å)	1.07		0.08
R.M.S.D. *H. orenii* (Å)			1.07

^a^Sequence corrected by changing Val^162^ to Asp, according to^22^.

^b^Homology model performed using the SWISSMODEL^60^ server and the *Ea*BglA structure as a template.

**Table 2:** Molecular dynamics simulations of *Ho*BglA and *Ea*BglA biological units.

Enzyme	*Ho*BglA monomer	*Ea*BglA tetramer
MD simulations (10 °C/30 °C)^a^		
Mean RMSF (backbone) (Å)	0.47 (±0.18)/0.49 (±0.26)	0.48 (±0.21)/0.55 (±0.29)
Total H bonds	869/963	842/907^b^
Unstable H bonds (persistence <5%)	531/649	524/597^b^
Average H bonds per frame	351 (±6)/343 (±6)	341 (±3)/339 (±4)^b^
Average H bonds persistence	40%/36%	41%/37%
Total salt bridges	59/59	37/38^b^
Average salt bridge per frame	44 (±1.4)/43 (±1.3)	29(±0.5) /29 (±0.6)^b^
Average salt bridge persistence	73%/72%	76%/76%
Average total SAS (Å^2^)	17341 (±105)/17566 (±146)	15847 (±80)/15820 (±67)^b^
Average hydrophobic SAS (Å^2^)	3440 (±83)/3664 (±77)	4017 (±35)/4012 (±49)^b^
Hydrophobic SAS/Total SAS ratio	20%/21%	25%/25%

^a^The values were calculated from the last 80 ns data of 100 ns simulations and are presented in the form 10 °C/30 °C.

^b^The data calculated for the tetramer were divided by four to represent the values per chain.

**Table 3:** Data collection and refinement statistics.

Crystal form	I	II
Data collection		
Space group	*P*2_1_	*C*222_1_
Cell dimensions		
* a*, *b*, *c* (Å)	110.07, 104.60, 199.19	115.97, 376.12, 109.49
* α, β, γ* (°)	90, 105.8, 90	90
Molecules per AU^a^	8	4
Resolution (Å)	50.00–2.24 (2.38–2.24)^b^	50.00–2.15 (2.28–2.15)
Observed reflections	792,790 (115,412)	776,096 (90,946)
Unique reflections	203,452 (29,242)	250,365 (39,185)
*R*_merge_ (%)	10.3 (77.0)	8.6 (75.6)
*R*_meas_ (%)	11.9 (89.2)	10.3 (96.5)
*<I*/σ*I>*	9.5 (1.7)	8.2 (1.2)
CC_1/2_^c^	0.99 (0.73)	0.99 (0.69)
Completeness (%)	97.4 (87.0)	99.0 (96.0)
Multiplicity	3.9 (3.9)	3.1 (2.3)
Refinement		
Resolution (Å)	49.11–2.24	20.00–2.15
No. reflections	190,890	123,301
*R*_work_/*R*_free_	0.21/0.23	0.19/0.22
No. atoms		
* *Protein	28,766	14,456
* *Water/ligand	152	640
Mean *B*-factors (Å^2^)		
* *Protein	44.2	44.1
* *Water/ion	57.8	53.4
R.m.s. deviations		
* *Bond lengths (Å)	0.007	0.011
* *Bond angles (°)	1.103	1.360
Ramachandran Plot		
* *Favored (%)	96.6	97.1
* *Allowed (%)	3.4	2.9
* *Disallowed (%)	0.0	0.0
Molprobity clashscore	0.96	0.78

^a^AU, asymmetric unit.

^b^Values in parentheses are for highest-resolution shell.

^c^CC_1/2_, correlation between intensities from random half-datasets^61^.
