# Supplementary material for: Oligomerization as a strategy for cold adaptation: Structure and dynamics of the GH1 β-glucosidase from Exiguobacterium antarcticum B7
Source: Sci Rep. 2016 Mar 31;6:23776. doi: 10.1038/srep23776 (PMC4815018; doi:10.1038/srep23776)
Supplement: Supplementary Information [file srep23776-s1.pdf]

Supplementary material

Oligomerization as a strategy for cold adaptation: Structure and dynamics of the GH1 β-glucosidase from *Exiguobacterium antarcticum* B7

Leticia Maria Zanphorlin[1,#], Priscila Oliveira de Giuseppe[2,#], Rodrigo Vargas Honorato[2], Celisa Caldana Costa Tonoli[2], Juliana Fattori[2], Elaine Crespim[1], Paulo Sergio Lopes de Oliveira[2], Roberto Ruller[1], Mario Tyago Murakami[2,*]

Table S1. *In* solution and crystal parameters of *Ea*BglA tetramer.

**Parameters**	**AUC**	**SAXS**	**XTAL**[b]
D_{max} (Å)	-	115	119
R_g (Å)	-	36.00±0.01[a]	38.75
MW (kDa)	206±11	-	203.5
$s^0_{20,w}$ (S)	7.0±0.1	-	-

[a]Guinier analysis

[b]Crystallographic tetramer

Table S2. Active site volume and tetramer interface area parameters calculated for the two tetramers observed in the asymmetric unit of the *P*2$_1$ crystal (T_1 and T_2) and the two tetramers retrieved by symmetry relationships in the *C*222$_1$ crystal (T_3 and T_4) using KVfinder[1] and EPPIC[2], respectively.

**Chain**	**Active site volume (Å^3)**			
	T_1 (*P*2$_1$)	T_2 (*P*2$_1$)	T_3 (*C*222$_1$)	T_4 (*C*222$_1$)
A	475.20	491.20	504.79	510.19
B	495.94	496.37	516.89	511.92
C	525.10	514.51	657.50	670.90
D	650.16	680.18	675.43	671.76
**Interface**	**Interface area (Å^2)**			
	T_1 (*P*2$_1$)	T_2 (*P*2$_1$)	T_3 (*C*222$_1$)	T_4 (*C*222$_1$)
α_1	863.50	879.52	888.35	869.65
α_2	899.34	888.77	894.17	869.65
γ_1	570.12	567.72	550.90	553.33
γ_2	575.26	576.09	550.90	565.08

Table S3. List of GH1 enzymes with known structures according to the CAZY[3] database and their respective quaternary structures reported at the PDB files.

**Archaea**			
**Protein Name**	**Specie**	**Quaternary structure**	**PDB accession code**
β-glycosidase (β-Gly;ASAC_1390)	*Acidilobus saccharovorans*	tetramer	4HA3, 4HA4
β-glucosidase / β-glycosidase / β-rutinosidase (Cel1A;CelB)	*Pyrococcus furiosus*	monomer/dimer/tetramer	3WQ8, 3APG, 3WDP
alkyl β-glucosidase (BGPh;BglA)	*Pyrococcus horikoshii*	monomer	1VFF
β-glycosidase S (LacS;Ss-β-gly)	*Sulfolobus solfataricus*	monomer/tetramer	1GOW, 1UWI, 1UWQ, 1UWR, 1UWS, 1UWT, 1UWU, 2CEQ, 2CER, 4EAM, 4EAN
β-glycosidase (Tα-β-gly)	*Thermosphaera aggregans*	tetramer	1QVB
**Bacteria**			
**Protein Name**	**Specie**	**Quaternary structure**	**PDB accession code**
β-glucosidase (BglA)	*Bacillus circulans subsp. alkalophilus*	octamer	1QOX
β-glucosidase A (BglA;BG)	*Clostridium cellulovorans*	monomer	3AHX
6-P-β-glucosidase A (BglA;b2901)	*Escherichia coli str. K-12*	dimer	2XHY
β-glucosidase A (BglA;HoBGLA)	*Halothermothrix orenii*	monomer	3TA9, 4PTV, 4PTW, 4PTX
6-P-β-glucosidase (Pbg1;LpPbg1)	*Lactobacillus plantarum*	dimer	3QOM, 4GZE
6-P-β-galactosidase (LacG)	*Lactococcus lactis*	monomer	1PBG, 2PBG, 3PBG, 4PBG
β-glycosidase (BGL167;BglG167c)	*Microbacterium sp. Gsoil167*	dimer	4R27
β-glucosidase (Bgl;BglU)	*Micrococcus antarcticus*	monomer	3W53
β-glucosidase A (BglA)	*Paenibacillus polymyxa*	octamer	1BGA, 1BGG, 1E4I, 1TR1, 1UYQ
β-glucosidase B (BglB)	*Paenibacillus polymyxa*	monomer	2JIE, 2O9P, 2O9R, 2O9T, 2Z1S
6-P-β-glucosidase (CelA;Bgl)	*Streptococcus mutans*	monomer/dimer	3PN8, 4DDE, 4F66, 4F79, 4GPN
6-P-β-glucosidase (BglA-2;SP0578)	*Streptococcus pneumoniae*	dimer	4IPL, 4IPN
6-phospho-β-glucosidase (SPy_1599;SPy1599)	*Streptococcus pyogenes*	monomer	4B3K, 4B3L
β-glucosidase (Bgl3)	*Streptomyces sp.*	monomer/dimer	1GNX, 1GON
β-glucosidase A (BglA;Tmari_1862)	*Thermotoga maritima*	monomer	1OD0, 1OIF, 1OIM, 1OIN, 1UZ1, 1W3J, 2CBU, 2CBV, 2CES, 2CET, 2J75, 2J77, 2J78, 2J79, 2J7B, 2J7C, 2J7D, 2J7E, 2J7F, 2J7G, 2J7H, 2JAL, 2VRJ, 2WBG, 2WC3, 2WC4
β-glycosidase (Gly)	*Thermus nonproteolyticus*	monomer	1NP2

β-glycosidase / β-glucosidase (TTHB087)	*Thermus thermophilus*	monomer	1UG6, 4BCE
β-glycosidase (BglT;B-gly)	*Thermus thermophilus*	monomer	3ZJK
β-glucosidase / β-glycosidase (BglA;Bgl1A)	*uncultured bacterium*	monomer	3CMJ, 3FIY, 3FIZ, 3FJ0, 4HZ6, 4HZ7, 4HZ8
**Eukaryota**			
**Protein Name**	**Specie**	**Quaternary structure**	**PDB accession code**
myrosinase (BMY1)	*Brevicoryne brassicae*	monomer	1WCG
β-primeverosidase (Bgl;PD)	*Camellia sinensis*	monomer	3WQ4, 3WQ5, 3WQ6
neutral β-glycosylceramidase / β-glucosidase (Gba3;CbgCBgl1)	*Homo sapiens*	monomer	2E9L, 2E9M, 2JFE, 2ZOX, 3VKK
β-glucosidase (Bgl4;Bglhi)	*Humicola insolens*	monomer	4MDO, 4MDP
β-glucosidase (Bgl;NkBgl;NkBG)	*Neotermes koshunensis*	monomer	3AHZ, 3AI0, 3VIF, 3VIG, 3VIH, 3VII, 3VIJ, 3VIK, 3VIL, 3VIM, 3VIN, 3VIO, 3VIP
β-mannosidase (Os7BGlu26)	*Oryza sativa Indica Group*	monomer	4JHO, 4JIE
β-glucosidase (Os03g0212800;Os3bglu6)	*Oryza sativa Japonica Group*	monomer	3GNO, 3GNP, 3GNR, 3WBA, 3WBE
β-glucosidase (OsTAGG2;Os4bglu12)	*Oryza sativa Japonica Group*	monomer	3PTK, 3PTM, 3PTQ
β-glucosidase (Bglu1;Os3bglu7)	*Oryza sativa Japonica Group*	monomer	2RGL, 2RGM, 3AHT, 3AHV, 3F4V, 3F5I, 3F5J, 3F5K, 3F5L, 3SCN, 3SCO, 3SCP, 3SCQ, 3SCR, 3SCS, 3SCT, 3SCU, 3SCV, 3SCW, 4QLJ, 4QLK, 4QLL
β-glucosidase (Bgl1A)	*Phanerochaete chrysosporium*	monomer	2E3Z, 2E40
strictosidine β-glucosidase (Sgr1;SG)	*Rauvolfia serpentina*	monomer/octamer	2JF6, 2JF7, 3ZJ7, 3ZJ8
raucaffricine O-β-glucosidase (RG)	*Rauvolfia serpentina*	monomer/dimer/octamer	3U57, 3U5U, 3U5Y, 3ZJ6, 4A3Y, 4ATD, 4ATL, 4EK7
β-glucosidase (ScGlu)	*Secale cereale*	hexamer	3AIU, 3AIV, 3AIW
myrosinase (partial)	*Sinapis alba*	monomer/dimer	1DWA, 1DWF, 1DWG, 1DWH, 1DWI, 1DWJ, 1E4M, 1E6Q, 1E6S, 1E6X, 1E70, 1E71, 1E72, 1E73, 1MYR, 1W9B, 1W9D, 2MYR, 2WXD
cyanogenic β-glucosidase / dhurrinase 1 (Dhr1)	*Sorghum bicolor*	dimer	1V02, 1V03
β-glucosidase 2 (CEL1a;Bgl2;BGLII;BGL) (Cel1A)	*Trichoderma reesei*	monomer	3AHY
β-glucosidase 2 (cyanogenic)	*Trifolium repens*	dimer	1CBG
β-glucosidase (Glu1B;TaGlu1b)	*Triticum aestivum*	hexamer	2DGA, 3AIQ, 3AIR, 3AIS
β-glucosidase 1 (Glu1;p60.1;Zm-p60.1)	*Zea mays*	monomer/dimer	1HXJ, 1E1E, 1E1F, 1E4L, 1E4N, 1E55, 1E56, 1H49, 1V08
**unclassified**			
**Protein Name**	**Specie**	**Quaternary structure**	**PDB accession code**
β-glucosidase (Td2F2)	unidentified	monomer	3WH5, 3WH7, 3WH8, 3WH6

Table S4. Number of structurally characterized GH1 enzymes in relation to the oligomeric state reported in the PDB.

oligomeric state	number of enzymes	fraction
monomer	24	55%
dimer	10	23%
tetramer	4	9%
hexamer	2	5%
octamer	4	9%

Table S5. Classification of bacteria species shown in Fig. 7 as psychrotroph (minimal growth temperature < 5ºC; or habitat temperature < 20 ºC), psychrophile (optimal temperature < 20 ºC), mesophile (minimal growth temperature > 5 ºC; or optimal temperature 20 ºC < t > 40 ºC) and thermophile (minimal growth temperature or optimal temperature > 40 ºC). NI = not identified.

Specie	Growth temperature range or (optimal Temp.)	Classification	Reference
Exiguobacterium antarcticum	-3 to 42ºC	psychrotroph	4
Exiguobacterium marinum	15 to 42ºC	mesophile	4
Bacillus aquimaris	10 to 44ºC	mesophile	5
Bacillus aurantiacus	10 to 45ºC	mesophile	6
Pontibacillus yanchengensis	15 to 45ºC	mesophile	7
Colwellia psychrerythraea	(10 °C)	psychrophile	8
Colwellia piezophila	(10 °C)	psychrophile	9
Candidatus Thiomargarita nelsonii	*habitat* 12ºC	psychrotroph	10
Thalassomonas actiniarum	15 to 37ºC	mesophile	11
Desulfotalea psychrophila	(10ºC)	psychrophile	12
Desulfonatronum lacustre	(37ºC)	mesophile	13
Pseudoalteromonas haloplanktis	(15ºC)	psychrophile	14
Pseudoalteromonas marina	4 to 37ºC	psychrotroph	15
Pseudoalteromonas lipolytica	15 to 37ºC	mesophile	16
Pseudoalteromonas agarivorans	7 to 35 °C	mesophile	17
Octadecabacter antarcticus	4 to 10ºC	psychrotroph	18
Octadecabacter arcticus	4 to 15ºC	psychrophile	18
Nautella italica	4 to 45ºC	psychrotroph	19
Ruegeria conchae	10 to 37ºC	mesophile	20
Rhodobacter sphaeroides	(25ºC)	mesophile	21
Micrococcus antarcticus	(15ºC)	psychrophile	22
Arthrobacter arilaitensis	10 to 30ºC	mesophile	23
Leifsonia aquatica	7 to 37ºC	mesophile	24
Glaciibacter superstes	−5 to 25ºC	psychrotroph	25
Agromyces subbeticus	6 to 37ºC	mesophile	26
Cryobacterium sp. MLB-32	*habitat (glaciers)*	psychrotroph	27
Herbiconiux sp. YR403	NI	NI	

Microbacterium indicum	8 to 30°C	mesophile	[28]
Arthrobacter chlorophenolicus	3 to 37ºC	psychrotroph	[29]
Bacillus psychrosaccharolyticus	0 to 30ºC	psychrotroph	[30]
Gracilibacillus lacisalsi	15 to 50ºC	mesophile	[31]
Bacillus halodurans	15 to 55ºC	mesophile	[32]
Bacillus thermoamylovorans	(50ºC)	thermophile	[33]
Planococcus halocryophilus	−10 to 37ºC	psychrotroph	[34]
Bacillus bogoriensis	10 to 40ºC	mesophile	[35]
Halobacillus halophilus	(30ºC)	mesophile	[36]
Shewanella baltica	4 not 37ºC	psychrotroph	[37]
Shewanella denitrificans	4 to 30ºC	psychrotroph	[38]
Shewanella piezotolerans WP3	0 to 28°C	psychrotroph	[39]
Shewanella violacea DSS12	4 to 30ºC	psychrotroph	[40]
Shewanella woodyi ATCC 51908	4 to 25ºC	psychrotroph	[41]
Halothermothrix orenii	45 to 68ºC	thermophile	[42]
Halobacteroides halobius	(37ºC)	mesophile	[43]
Halanaerobium saccharolyticum	(37ºC)	mesophile	[44]
Caldicoprobacter oshimai	42 to 79ºC	thermophile	[45]
Caldanaerobius polysaccharolyticus	45 to 72ºC	thermophile	[46]

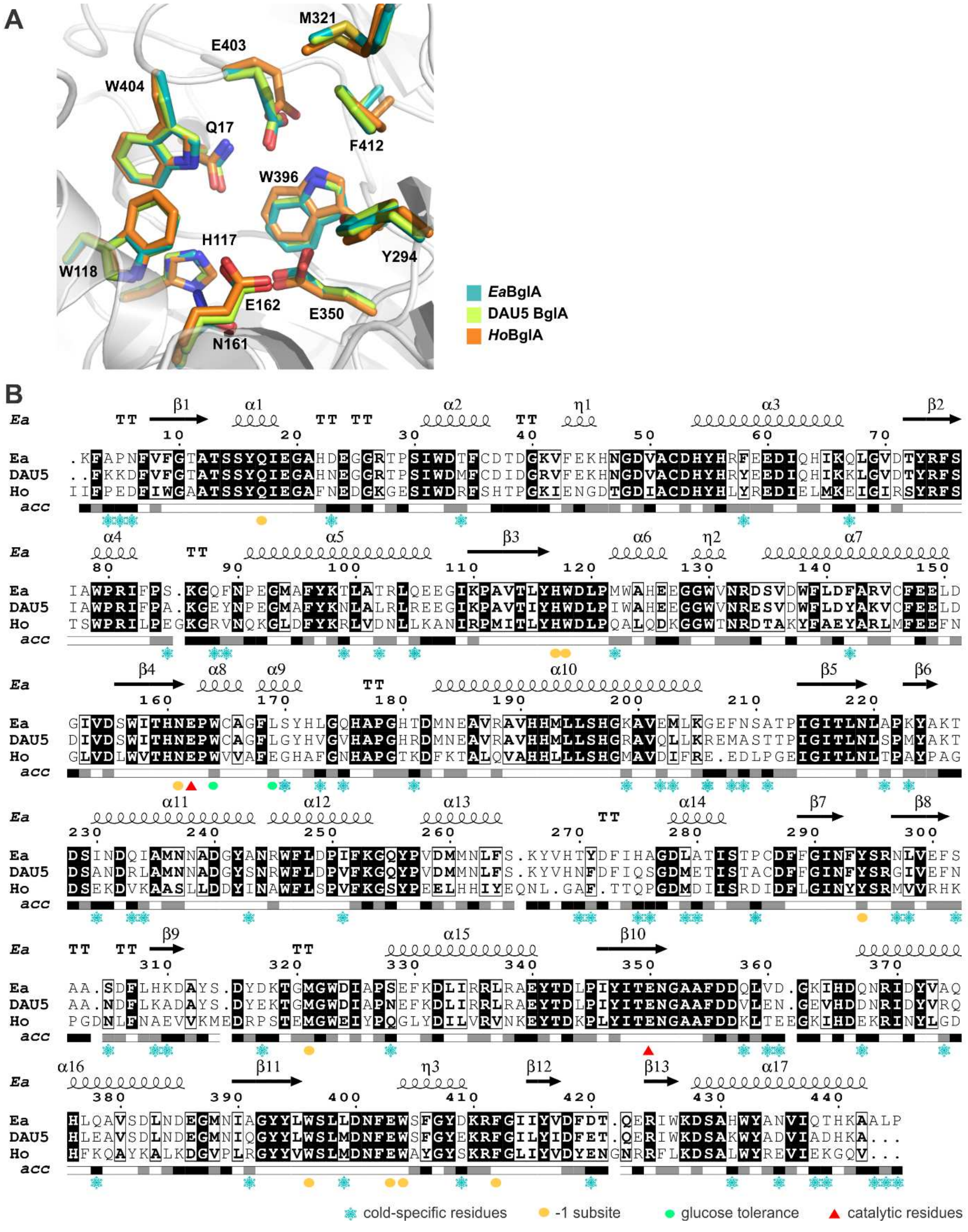

Figure S1. Structural comparison of the -1 subsite *(A)* and structure-based sequence alignment *(B)* between the β-glucosidases from *E. antarcticum (Ea), Exiguobacterium sp.* DAU5 strain (homology model constructed using SWISS-MODEL[47]) and *H. orenii* (PDB ID: 4PTX). Acc = solvent accessibility colored from white (buried) to black (exposed). Secondary structure elements are shown above the alignment and refer to the *Ea*BglA crystal structure. Picture was made using ESPript 3.0[48]. The residues only found in *Ea*BglA (blue asterisk), forming the -1 subsite (yellow sphere) or related to glucose tolerance (green sphere) are indicated by colored symbols. The catalytic residues are also marked with red triangles.

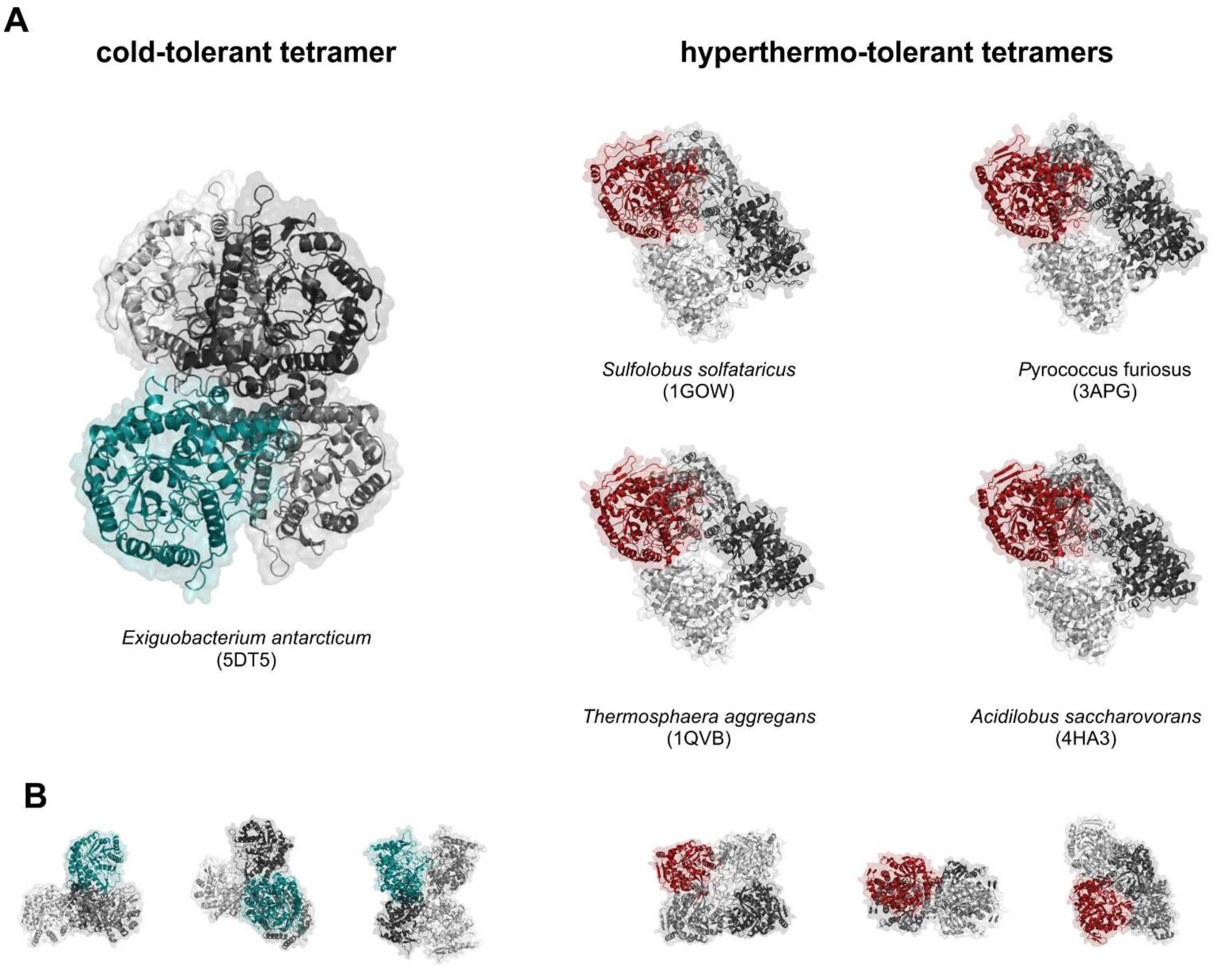

Figure S2. *Ea*BglA assumes a novel tetrameric arrangement compared to GH1 tetramers of known structure. *(A)* For comparison purposes, the *Ea*BglA protomer highlighted in blue is in the same orientation as the hyperthermophilic protomers shown in red. Other protomers are colored in different shades of grey. PDB codes are in parentheses. *(B)* Different orientations of the *Ea*BglA tetramer (blue/grey) and the correspondent views of a hyperthermo-tolerant tetramer (red/grey; PDB ID 4HA3) to evidence that they have distinct configurations.

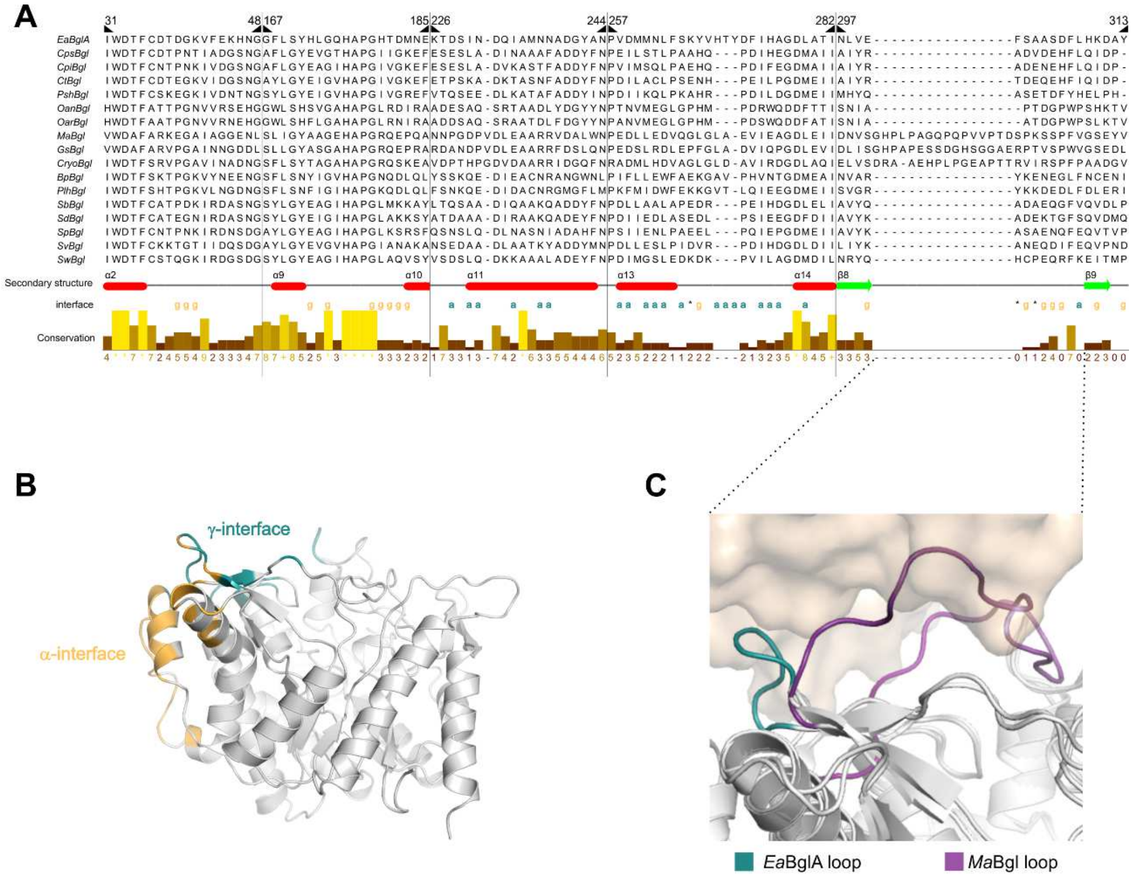

Figure S3. Sequence and structure comparison of the *Ea*BglA tetramer interfaces with other cold-adapted enzymes. *(A)* Protein sequence alignment truncated at the black arrows to show only regions containing residues from the α (green a), γ (yellow g) or both (*) interfaces that compose the tetramer. Numbers above the alignment indicate amino acid positions. Red bars represent α-helices, green arrows indicate β-sheets and the grey line indicate loops. Position conservation is colored from brown (0 = absent) to yellow (* = fully conserved). Species names and the NCBI accession codes of each protein sequence are as follows: *Cps* = *Colwellia psychrerythraea* (WP 011044459.1); *Cpi* = *C. piezophila* (WP_019026132.1); *Ct* = *Candidatus Thiomargarita nelsonii* (KHD12531.1); *Psh* = *Pseudoalteromonas haloplanktis* (WP 002961837.1); *Oan* = *Octadecabacter antarcticus* (WP 015499963.1); *Oar* = *O. arcticus* (WP 015494812.1)*; Ma* = *Micrococcus antarcticus* (ACM66669.1)*; Gs* = *Glaciibacter superstes* (WP 026851108.1); *Cryo* = *Cryobacterium sp. MLB-32* (WP 035875375.1)*; Bp* = *Bacillus psychrosaccharolyticus* (WP 040376320.1); *Plh* = *Planococcus halocryophilus* (WP 040850804.1); *Sb* = *Shewanella baltica* OS155 (ABN60651.1)*; Sd* = *S. denitrificans* (WP 011497037.1); *Sp* = *S. piezotolerans* WP3 (ACJ28412.1)*; Sv* = *S. violacea* DSS12 (BAJ01494.1)*; Sw* = *S. woodyi* ATCC 51908 (ACA85440.1). *(B) Ea*BglA protomer highlighting the location of the α and γ interfaces. (C) Structural comparison between *Ea*BglA and *Ma*Bgl (PDB ID: 3W53) crystal structures highlighting the β8/β9 loop, which is 16 residues longer in *Ma*Bgl and cause an steric hindrance to the formation of the γ interface with a neighbor protomer (yellow surface, *Ea*BglA subunit).

Supplementary references

1 Oliveira, S. H. *et al.* KVFinder: steered identification of protein cavities as a PyMOL plugin. *BMC bioinformatics* **15**, 197, doi:10.1186/1471-2105-15-197 (2014).

2 Duarte, J. M., Srebniak, A., Scharer, M. A. & Capitani, G. Protein interface classification by evolutionary analysis. *BMC bioinformatics* **13**, 334, doi:10.1186/1471-2105-13-334 (2012).

3 Henrissat, B. A classification of glycosyl hydrolases based on amino acid sequence similarities. *The Biochemical journal* **280 (Pt 2)**, 309-316 (1991).

4 Vishnivetskaya, T. A., Kathariou, S. & Tiedje, J. M. The Exiguobacterium genus: biodiversity and biogeography. *Extremophiles : life under extreme conditions* **13**, 541-555, doi:10.1007/s00792-009-0243-5 (2009).

5 Yoon, J. H., Kim, I. G., Kang, K. H., Oh, T. K. & Park, Y. H. Bacillus marisflavi sp. nov. and Bacillus aquimaris sp. nov., isolated from sea water of a tidal flat of the Yellow Sea in Korea. *International journal of systematic and evolutionary microbiology* **53**, 1297-1303 (2003).

6 Borsodi, A. K. *et al.* Bacillus aurantiacus sp. nov., an alkaliphilic and moderately halophilic bacterium isolated from Hungarian soda lakes. *International journal of systematic and evolutionary microbiology* **58**, 845-851, doi:10.1099/ijs.0.65325-0 (2008).

7 Yang, Y., Zou, Z., He, M. & Wang, G. Pontibacillus yanchengensis sp. nov., a moderately halophilic bacterium isolated from salt field soil. *International journal of systematic and evolutionary microbiology* **61**, 1906-1911, doi:10.1099/ijs.0.023911-0 (2011).

8 Deming, J. W., Somers, L. K., Straube, W. L., Swartz, D. G. & Macdonell, M. T. Isolation of an Obligately Barophilic Bacterium and Description of a New Genus, Colwellia gen. nov. *Systematic and Applied Microbiology* **10**, 152-160 (1988).

9 Nogi, Y., Hosoya, S., Kato, C. & Horikoshi, K. Colwellia piezophila sp. nov., a novel piezophilic species from deep-sea sediments of the Japan Trench. *International journal of systematic and evolutionary microbiology* **54**, 1627-1631, doi:10.1099/ijs.0.03049-0 (2004).

10 Grünke, S. *et al.* Mats of psychrophilic thiotrophic bacteria associated with cold seeps of the Barents Sea. *Biogeosciences* **9**, 2947-2960, doi:10.5194/bg-9-2947-2012 (2012).

11 Hosoya, S., Adachi, K. & Kasai, H. Thalassomonas actiniarum sp. nov. and Thalassomonas haliotis sp. nov., isolated from marine animals. *International journal of systematic and evolutionary microbiology* **59**, 686-690, doi:10.1099/ijs.0.000539-0 (2009).

12 Knoblauch, C., Sahm, K. & Jorgensen, B. B. Psychrophilic sulfate-reducing bacteria isolated from permanently cold arctic marine sediments: description of Desulfofrigus oceanense gen. nov., sp. nov., Desulfofrigus fragile sp. nov., Desulfofaba gelida gen. nov., sp. nov., Desulfotalea psychrophila gen. nov., sp. nov. and Desulfotalea arctica sp. nov. *International journal of systematic bacteriology* **49 Pt 4**, 1631-1643 (1999).

13 PIKUTA *et al.* Vol. 67 (Springer, New York, NY, ETATS-UNIS, 1998).

14 Corsaro, M. M., Lanzetta, R., Parrilli, E., Parrilli, M. & Tutino, M. L. Structural investigation on the lipooligosaccharide fraction of psychrophilic Pseudoalteromonas haloplanktis TAC 125 bacterium. *European journal of biochemistry / FEBS* **268**, 5092-5097 (2001).

15 Nam, Y. D. *et al.* Pseudoalteromonas marina sp. nov., a marine bacterium isolated from tidal flats of the Yellow Sea, and reclassification of Pseudoalteromonas sagamiensis as Algicola sagamiensis comb. nov. *International journal of systematic and evolutionary microbiology* **57**, 12-18, doi:10.1099/ijs.0.64523-0 (2007).

16 Xu, X. W. *et al.* Pseudoalteromonas lipolytica sp. nov., isolated from the Yangtze River estuary. *International journal of systematic and evolutionary microbiology* **60**, 2176-2181, doi:10.1099/ijs.0.017673-0 (2010).

17 Romanenko, L. A. *et al.* Pseudoalteromonas agarivorans sp. nov., a novel marine agarolytic bacterium. *International journal of systematic and evolutionary microbiology* **53**, 125-131 (2003).

18 Gosink, J. J., Herwig, R. P. & Staley, J. T. Octadecabacter arcticus gen. nov., sp. nov., and O. antarcticus, sp. nov., Nonpigmented, Psychrophilic Gas Vacuolate Bacteria from Polar Sea Ice and Water. *Systematic and Applied Microbiology* **20**, 356-365 (1997).

19 Vandecandelaere, I. *et al.* Nautella italica gen. nov., sp. nov., isolated from a marine electroactive biofilm. *International journal of systematic and evolutionary microbiology* **59**, 811-817, doi:10.1099/ijs.0.002683-0 (2009).
20 Lee, J. *et al.* Ruegeria conchae sp. nov., isolated from the ark clam Scapharca broughtonii. *International journal of systematic and evolutionary microbiology* **62**, 2851-2857, doi:10.1099/ijs.0.037283-0 (2012).
21 Curators. (ed Leibniz Institut DSMZ-Deutsche Sammlung von Mikroorganismen und Zellkulturen GmbH) DSM 2340.
22 Liu, H., Xu, Y., Ma, Y. & Zhou, P. Characterization of Micrococcus antarcticus sp. nov., a psychrophilic bacterium from Antarctica. *International journal of systematic and evolutionary microbiology* **50 Pt 2**, 715-719 (2000).
23 Irlinger, F., Bimet, F., Delettre, J., Lefevre, M. & Grimont, P. A. Arthrobacter bergerei sp. nov. and Arthrobacter arilaitensis sp. nov., novel coryneform species isolated from the surfaces of cheeses. *International journal of systematic and evolutionary microbiology* **55**, 457-462, doi:10.1099/ijs.0.63125-0 (2005).
24 Evtushenko, L. I., Dorofeeva, L. V., Subbotin, S. A., Cole, J. R. & Tiedje, J. M. Leifsonia poae gen. nov., sp. nov., isolated from nematode galls on Poa annua, and reclassification of 'Corynebacterium aquaticum' Leifson 1962 as Leifsonia aquatica (ex Leifson 1962) gen. nov., nom. rev., comb. nov. and Clavibacter xyli Davis et al. 1984 with two subspecies as Leifsonia xyli (Davis et al. 1984) gen. nov., comb. nov. *International journal of systematic and evolutionary microbiology* **50 Pt 1**, 371-380 (2000).
25 Katayama, T. *et al.* Glaciibacter superstes gen. nov., sp. nov., a novel member of the family Microbacteriaceae isolated from a permafrost ice wedge. *International journal of systematic and evolutionary microbiology* **59**, 482-486, doi:10.1099/ijs.0.001354-0 (2009).
26 Jurado, V., Groth, I., Gonzalez, J. M., Laiz, L. & Saiz-Jimenez, C. Agromyces subbeticus sp. nov., isolated from a cave in southern Spain. *International journal of systematic and evolutionary microbiology* **55**, 1897-1901, doi:10.1099/ijs.0.63637-0 (2005).
27 Singh, P., Singh, S. M. & Dhakephalkar, P. Diversity, cold active enzymes and adaptation strategies of bacteria inhabiting glacier cryoconite holes of High Arctic. *Extremophiles : life under extreme conditions* **18**, 229-242, doi:10.1007/s00792-013-0609-6 (2014).
28 Shivaji, S. *et al.* Microbacterium indicum sp. nov., isolated from a deep-sea sediment sample from the Chagos Trench, Indian Ocean. *International journal of systematic and evolutionary microbiology* **57**, 1819-1822, doi:10.1099/ijs.0.64782-0 (2007).
29 Westerberg, K., Elvang, A. M., Stackebrandt, E. & Jansson, J. K. Arthrobacter chlorophenolicus sp. nov., a new species capable of degrading high concentrations of 4-chlorophenol. *International journal of systematic and evolutionary microbiology* **50 Pt 6**, 2083-2092 (2000).
30 Larkin, J. M. & Stokes, J. L. Isolation of psychrophilic species of Bacillus. *Journal of bacteriology* **91**, 1667-1671 (1966).
31 Jeon, C. O. *et al.* Gracilibacillus lacisalsi sp. nov., a halophilic Gram-positive bacterium from a salt lake in China. *International journal of systematic and evolutionary microbiology* **58**, 2282-2286, doi:10.1099/ijs.0.65369-0 (2008).
32 Nielsen, P., Fritze, D. & Priest, F. G. Phenetic diversity of alkaliphilic Bacillus strains: proposal for nine new species. *Microbiology* **141**, 1745-1761, doi:doi:10.1099/13500872-141-7-1745 (1995).
33 Combet-Blanc, Y. *et al.* Bacillus thermoamylovorans sp. nov., a moderately thermophile and amylolytic bacterium. *International journal of systematic bacteriology* **45**, 9-16 (1995).
34 Mykytczuk, N. C., Wilhelm, R. C. & Whyte, L. G. Planococcus halocryophilus sp. nov., an extreme sub-zero species from high Arctic permafrost. *International journal of systematic and evolutionary microbiology* **62**, 1937-1944, doi:10.1099/ijs.0.035782-0 (2012).
35 Vargas, V. A., Delgado, O. D., Hatti-Kaul, R. & Mattiasson, B. Bacillus bogoriensis sp. nov., a novel alkaliphilic, halotolerant bacterium isolated from a Kenyan soda lake. *International journal of systematic and evolutionary microbiology* **55**, 899-902, doi:10.1099/ijs.0.63318-0 (2005).
36 Curators. (ed Leibniz Institut DSMZ-Deutsche Sammlung von Mikroorganismen und Zellkulturen GmbH) DSM 2266.

37 Ziemke, F., Hofle, M. G., Lalucat, J. & Rossello-Mora, R. Reclassification of Shewanella putrefaciens Owen's genomic group II as Shewanella baltica sp. nov. *International journal of systematic bacteriology* **48 Pt 1**, 179-186 (1998).

38 Brettar, I., Christen, R. & Hofle, M. G. Shewanella denitrificans sp. nov., a vigorously denitrifying bacterium isolated from the oxic-anoxic interface of the Gotland Deep in the central Baltic Sea. *International journal of systematic and evolutionary microbiology* **52**, 2211-2217 (2002).

39 Xiao, X., Wang, P., Zeng, X., Bartlett, D. H. & Wang, F. Shewanella psychrophila sp. nov. and Shewanella piezotolerans sp. nov., isolated from west Pacific deep-sea sediment. *International journal of systematic and evolutionary microbiology* **57**, 60-65, doi:10.1099/ijs.0.64500-0 (2007).

40 Kato, C. & Nogi, Y. Correlation between phylogenetic structure and function: examples from deep-sea Shewanella. *FEMS microbiology ecology* **35**, 223-230 (2001).

41 Makemson, J. C. *et al.* Shewanella woodyi sp. nov., an exclusively respiratory luminous bacterium isolated from the Alboran Sea. *International journal of systematic bacteriology* **47**, 1034-1039 (1997).

42 Cayol, J. L. *et al.* Isolation and characterization of Halothermothrix orenii gen. nov., sp. nov., a halophilic, thermophile, fermentative, strictly anaerobic bacterium. *International journal of systematic bacteriology* **44**, 534-540 (1994).

43 Oren, A., Weisburg, W. G., Kessel, M. & Woese, C. R. Halobacteroides halobius gen. nov., sp. nov., a Moderately Halophic Anaerobic Bacterium from the Bottom Sediments of the Dead Sea. *Systematic and Applied Microbiology* **5**, 58-70 (1984).

44 Curators. (ed Leibniz Institut DSMZ-Deutsche Sammlung von Mikroorganismen und Zellkulturen GmbH) DSM 6641.

45 Yokoyama, H., Wagner, I. D. & Wiegel, J. Caldicoprobacter oshimai gen. nov., sp. nov., an anaerobic, xylanolytic, extremely thermophile bacterium isolated from sheep faeces, and proposal of Caldicoprobacteraceae fam. nov. *International journal of systematic and evolutionary microbiology* **60**, 67-71, doi:10.1099/ijs.0.011379-0 (2010).

46 Cann, I. K., Stroot, P. G., Mackie, K. R., White, B. A. & Mackie, R. I. Characterization of two novel saccharolytic, anaerobic thermophiles, Thermoanaerobacterium polysaccharolyticum sp. nov. and Thermoanaerobacterium zeae sp. nov., and emendation of the genus Thermoanaerobacterium. *International journal of systematic and evolutionary microbiology* **51**, 293-302 (2001).

47 Schwede, T., Kopp, J., Guex, N. & Peitsch, M. C. SWISS-MODEL: An automated protein homology-modeling server. *Nucleic acids research* **31**, 3381-3385 (2003).

48 Robert, X. & Gouet, P. Deciphering key features in protein structures with the new ENDscript server. *Nucleic acids research* **42**, W320-324, doi:10.1093/nar/gku316 (2014).
